# Polycyclic aromatic hydrocarbons produced by electrocautery smoke and the use of personal protective equipment **^1^**

**DOI:** 10.1590/1518-8345.1561.2853

**Published:** 2017-03-09

**Authors:** Caroline Vieira Claudio, Renata Perfeito Ribeiro, Júlia Trevisan Martins, Maria Helena Palucci Marziale, Maria Cristina Solci, José Carlos Dalmas

**Affiliations:** 2 MSc, RN, Hospital do Coração de Londrina, Londrina, PR, Brazil.; 3 PhD, Adjunct Professor, Departamento de Enfermagem, Universidade Estadual de Londrina, Londrina, PR, Brazil.; 4 PhD, Full Professor, Escola de Enfermagem de Ribeirão Preto da Universidade de São Paulo, WHO Collaborating Centre for Nursing Research Development, Ribeirão Preto, SP, Brazil.; 5 PhD, Associate Professor, Departamento de Química, Universidade Estadual de Londrina, Londrina, PR, Brazil.; 6 PhD, Associate Professor, Departamento de Estatística, Universidade Estadual de Londrina, Londrina, PR, Brazil.

**Keywords:** Occupational Exposure, Air Pollutants, Occupational, Electrosurgery, Electrocoagulation, Protective Devices

## Abstract

**Objective:**

analyze the concentration of polycyclic aromatic hydrocarbons in electrocautery smoke in operating rooms and the use of personal protective equipment by the intraoperative team when exposed to hydrocarbons.

**Method:**

exploratory and cross-sectional field research conducted in a surgery center. Gases were collected by a vacuum suction pump from a sample of 50 abdominal surgeries in which an electrocautery was used. A form was applied to identify the use of personal protective equipment. Gases were analyzed using chromatography. Descriptive statistics and Spearman's test were used to treat data.

**Results:**

there were 17 (34%) cholecystectomies with an average duration of 136 minutes, while the average time of electrocautery usage was 3.6 minutes. Airborne hydrocarbons were detected in operating rooms in 100% of the surgeries. Naphthalene was detected in 48 (96.0%) surgeries and phenanthrene in 49 (98.0%). The average concentration of these compounds was 0.0061 mg/m3 and a strong correlation (0.761) was found between them. The intraoperative teams did not use respirator masks such as the N95.

**Conclusion:**

electrocautery smoke produces gases that are harmful to the health of the intraoperative team, which is a concern considering the low adherence to the use of personal protective equipment.

## Introduction

The work environment in surgery centers (SC) is surrounded by occupational hazards due to peculiarities of the environment and the tasks performed there. Among these, we selected the chemical risk arising from the surgical smoke that results from electrocautery, a process used to dissect and coagulate tissues. Its use decreases surgical time and intraoperative bleeding^1^.

Electrocautery produces surgical smoke because tissues are heated. This smoke may contain various chemical pollutants in the form of gases or particles^2^. Exploratory studies report that polycyclic aromatic hydrocarbons (PAHs)^3^ ^-^ ^4^, volatile organic compounds^5^, and carbon oxides are among such pollutants contained in the smoke combustion process^2^ ^,^ ^6^.

These chemical compounds may have harmful effects on the human body, including cancer^7^ ^-^ ^8^ and respiratory symptoms, such as pharyngeal burning, nasal congestion, nausea and headache**^1^** **^-^** **^2^** **^)^** . Hence, the smoke generated by electrocautery represents a chemical hazard to the health of workers composing the intraoperative team.

PAHs are classified as organic chemical compounds containing at least two aromatic rings formed by carbon and hydrogen only^9^. These compounds are generated during incomplete combustion processes, such as those of coal, wood, garbage, tobacco, and grilled meat^8^, and are also formed during electrocautery. Studies developed in Lübeck (Germany)^3^, Changhua (Taiwan)^4^ and in Uppsala (Sweden)^10^ identified these compounds in the surgical smoke generated by this device.

Even though there are more than 100 types of PAHs^8^, the existing literature provides no recommendation regarding permissible exposure limits to any of these types. According to an American agency^11^ ^-^ ^12^, the occupational exposure limit value of naphthalene is 50 mg/m^3^, while the limit of anthracene, phenanthrene and pyrene is 0.2 mg/m^3^ each, considering occupational exposure on an eight-hour workday, on average. Nonetheless, the occupational exposure limits of these compounds have not been established for intraoperative teams working in operating rooms for different periods of time. 

Approximately 500,000 health workers in the United States, including surgeons, perioperative nurses, anesthesiologists, and nurses, are exposed to surgical smoke^13^. Medical and nursing graduating students present in the operating room are also exposed. We did not identify in our bibliographical survey^(14)^ any Brazilian study addressing the exposure to and composition of surgical smoke.

Preventive measures should be implemented during surgeries in operating rooms to decrease chemical hazards related to surgical smoke exposure, such as local exhaust systems^15^ and effective ventilation systems^16^, in addition to the use of respirators by the intraoperative staff, such as the N95^17^, and safety goggles^16^.

Seeking to expand knowledge regarding the harmfulness of PAH chemical exposure, this study was conducted to answer the following questions: - What are the PAH airborne concentrations in operating rooms arising from the surgical smoke produced by electrocautery during a surgery? - Does the intraoperative staff use proper personal protective equipment (PPE) when exposed to PAHs?

This study's general objective was to analyze the concentration of PAHs arising from the smoke produced by electrocautery in operating rooms and the use of PPE by the intraoperative team when exposed to hydrocarbons. The specific objectives were to characterize the environment in operating rooms and identify PAH concentrations arising from the smoke produced by electrocautery during surgery.

## Method

This exploratory, cross-sectional field research with a quantitative approach was conducted in an SC of a public university hospital located in the north of Paraná, Brazil. There are 262 health workers and graduate students working in this SC: eight nurses, eight perioperative nursing residents, 16 nursing technicians, 23 nursing auxiliaries, 18 anesthesiologists, 13 anesthesiology residents, 83 surgeons, and 93 surgery residents. All of these are exposed to surgical smoke. 

This SC is composed of seven operating rooms in which 700 surgeries, from various medical specialties, are performed per month, on average. Elective surgeries are performed on weekdays from 7am to 5pm, while urgent and emergency surgeries are also performed on weekends, including nights and holidays. 

This study addressed an intentional sample composed of 50 abdominal surgeries in which electrocautery was used. These surgeries were chosen because of their being high frequency abdominal surgeries (from one to three times a day), in which electrocautery is used. An average of 30 surgeries of this specialty are performed per month.

Inclusion criteria were abdominal surgeries using electrocautery, except emergency open abdominal and videolaparoscopic surgeries, as these are procedures that require immediate surgical intervention and it would be difficult to set up the apparatus used to collect data in time. The data collection system was assembled daily and at every surgical event. Data were collected during the morning, afternoon, and evening shifts from April 22 to July 8, 2015.

A form was used to characterize the operating rooms and another to characterize the surgeries, the use of the electrocautery, surgical staff, and PPE use (masks and goggles). Both forms were developed according to data provided in the literature and assessed in regard to content and objectivity by three nurses who are researchers experienced in the fields of occupational health and perioperative care, who deemed the instruments to be appropriate for this study. A pretest was performed with both instruments for six abdominal surgeries. 

The assessment of PPE used by the intraoperative team considered surgical masks, respirators (N95) and goggles^15^ ^-^ ^17^.

A vacuum suction pump, ASF Thomas^(r)^ model D-82178 Puchhe im, was tested to collect PAHs. The test showed the pump was valid and reliable for suctioning airborne PAH in operating rooms. This pump includes a battery and a plastic extension to which cartridges, adapted from five-milliliter syringes, were connected for every surgical event.

Each cartridge is composed of an Amberlite(r) XAD4 resin, characterized as a polymeric adsorbent with large pores able to remove aromatic compounds, such as PAHs, from the air, and a filter to allow the passage of PAHs in gaseous form only, impeding the passage of compounds in particulate form or polypropylene foam. The foam enabled the fixation of XAD4 resin, impeding it from leaving through the tip of the cartridge.

The vacuum suction pump remained on during all the surgeries included in the sample, under a flow rate of 120 liters per hour, from the time the surgical site was opened until its closure. It was located at a fixed point at the height of the workers' breathing zone, specifically seven centimeters from the operative field.

PAHs were extracted from the XAD4 resin, concentrated and then analyzed by high efficiency liquid chromatography. This chromatographer is able to analyze 16 types of PAHs, namely: naphthalene, acenaphthene, acenaphthylene, fluorine, phenanthrene, anthracene, fluoranthene, pyrene, chrysene, benzo(a)anthracene, benzo(b)fluoranthene, benzo(k)fluoranthene, benzo(a) pyrene, dibenz(a,h)anthracene, benzo(ghi)perylene, and indene(1,2,3-cd) pyrene.

The preparation, extraction of the cartridges and PAHs readings were conducted by one Master's student and one PhD student from the chemistry field in an air compounds analysis laboratory.

Data collected with the forms and PAH concentrations were recorded in a spreadsheet in Excel^(r)^ 2010 through sequence double entry, organized and analyzed using the Statistical Package for Social Science^TM^ version 20.0. Descriptive statistics were used so that the frequency and percentage of categorical variables (type of surgery, time of surgery, mode of electrocautery use, staff characterization, PPE usage) were calculated. Mean, standard deviation, median, minimum and maximum values were calculated for the continuous variables (surgery duration, how long the electrocautery was used, electrocautery power, and PAH concentrations). The Shapiro-Wilk test was used to test the hypothesis of normality in the distribution of quantitative variables, which was not normal (p<0.01). The non-parametrical Spearman's correlation test was used for the continuous variables, with a level of significance at 0.05.

This study was approved by the hospital's Institutional Review Board in September 2014 (Registration No. 34232714.1.0000.5231) and conducted according to the ethical guidelines established by the National Ethics Committee.

## Results

The operating rooms presented different dimensions: three were 34.22 m^2^; two were 32.78 m^2^; and another two were 45.47m^2^. Each room had two doors and two air conditioning systems, one central and one individual, both located at the top, though there were no exhaust fans.

Of the 50 surgeries in the sample, 11 (22%) were cholecystectomies, seven (14%) were appendectomies, and six (12%) were cholecystectomies associated with cholangiography. Twenty seven (54%) of these were open surgeries and 23 (46%) were videolaparoscopic procedures; 32 (64%) were elective and 18 (36%) were urgent surgeries. Twenty seven (54%) out of the 50 surgeries were performed in the afternoon. The surgeries lasted 136 minutes on average, with a standard deviation of ±84 minutes and median of 113 minutes.

Monopolar electrocautery was used in the surgeries for an average of 3.6 minutes, with standard deviation of 3.8 minutes and median of 2.4 minutes. The average power used was 54.7 watts, with standard deviation of ± 23.7 watts. Most surgeries (66%) used the cut and coagulation modes.

In regard to PAHs, naphthalene and phenanthrene compounds were found in the air of the operating rooms. Naphthalene was found in 48 (96%) surgeries and phenanthrene was detected in 49 (98%) of the surgeries assessed.

Table 1 presents the concentrations of PAHs identified in the surgeries.

**Table 1:** Mean and minimum and maximum values of PAH concentrations detected during surgical events (n=50). PR, Brazil, 2016

PAH variables* [mg/m³]	n (%)	Mean ± standard deviation	Minimum values	Maximum values
Naphthalene and/or phenanthrene	50 (100)	0.0061 ± 0.0049	0.0006	0.0208
Naphthalene	48 (96)	0.0053 ± 0.0043	0.0004	0.0188
Phenanthrene	49 (98)	0.0007 ± 0.0007	0.0001	0.0031

* PAH - polycyclic aromatic hydrocarbons

PAHs were found in all the samples (100%). The average concentrations of total PAHs (naphthalene and/or phenanthrene) were 0.0061 mg/m³, ranging from 0.0006 to 0.0208. In only two abdominal surgeries was naphthalene not found (cholecystectomy and videolaparoscopic appendectomy), while in only one surgery was phenanthrene not found (exploratory laparotomy associated with para-aortic lymph node biopsy). The average concentrations of these compounds were 0.0053 and 0.0007 for naphthalene and phenanthrene, respectively.

The Spearman's test revealed a correlation (0.761) between the naphthalene and phenanthrene variables.

In total, 62 health workers and graduate students were present in the surgeries: 11 (17.7%) nursing technicians and six (9.7%) auxiliaries; four (6.5%) perioperative nursing residents; six (9.7%) anesthesiologists; 11 (17.7%) anesthesiology residents; nine (14.5%) general surgeons; and 15 (24.2%) general surgery residents. Of these, 25 (40.3%) were females and 37 (59.7%) were males.

Table 2 presents the use of PPE by the perioperative team when exposed to the smoke produced by electrocautery in the 50 surgeries.

**Table 2:** Use of PPE by the intraoperative staff when exposed to smoke produced by electrocautery during surgeries. (n=62). PR, Brazil, 2016

Variables PPE use*	n=62			
	Yes				No	
	n	%		n	%
Respirator mask**^†^**	---	---		62	100
Surgical mask**^‡^**	56	90		6	10
Safety goggles	3	5		59	95

*PPE - Personal protective equipment; †Respirator masks such as the N95, are nationally and internationally recommended to prevent the inhalation of chemical compounds present in surgical smoke^15^ ^,^ ^17^; ‡Surgical masks, also known as procedure masks, are not recommended to protect against inhalation of surgical smoke^15^ ^,^ ^17^.

The measures recommended to minimize the effects of electrocautery usage include PPE. Respirator masks such as the N95 type filter at least 95% of aerosols, gases, and fumes^17^. The use of respirator masks is regulated by an American agency of occupational health as secondary protection to prevent the inhalation of surgical smoke^15^.

None of the workers or graduate students composing the intraoperative team used a respirator such as the N95. Most (90%) used a surgical mask during the surgical procedures, while six (10%) workers and graduates, among whom were five anesthesiology residents and one anesthetist, did not use even a surgical mask. Only three (5%) graduate students from the general surgery field wore protective goggles during electrocautery use.

## Discussion

The study's SC has one central and one individual air conditioning system; no exhausts exist, though. The literature emphasizes air treatment in health facilities, including ventilation and exhaust systems able to remove and filter airborne gases and microorganisms, decreasing chemical hazards^(18)^, as in the case of surgical smoke. The absence of exhausts in the SC in question, however, aggravates the quality of this environment's air, and consequently, poses risks to the health of the intraoperative team.

The air inlets and outlets should promote appropriate movement, always in the direction of the least to the most contaminated area. Additionally, air insufflation should be designed to minimize air currents and turbulence^(18)^. Air currents, which are often generated by an individual air-conditioning system, may facilitate the dispersion of PAHs in the air of the operating room.

In addition to recommending a ventilation system for the operating rooms, the American agency also establishes the use of a local exhaust/ventilation system, essential to removing gases from operating rooms that arise from surgical smoke, for both open surgical procedures and videolaparoscopic procedures. Local exhaust can be performed with a portable smoke evacuator containing a high efficiency filter for airborne compounds^15^.

Exploratory studies conducted in Lübeck (Germany), Uppsala (Sweden), Scotland (United Kingdom) and in Zurich (Switzerland) investigated chemical compounds coming out of surgical smoke and identified PAHs^3^ ^,^ ^6^ ^,^ ^10^ ^,^ ^19^ and volatile organic compounds^6^ ^,^ ^19^, both in open^10^ ^)^ and in videolaparoscopy procedures^6^.

The surgeries addressed in this study lasted 136 minutes on average, with a standard deviation of ±84 minutes, similar to the duration (143.4 minutes) reported by a study conducted with 15 abdominal surgeries and that also collected the surgical smoke from electrocautery^20^. The median found in this study, however, (113 minutes) was lower than the median found in another study assessing peritonectomies, the median of which was 614 minutes^10^.

The average time used for electrocautery was 3.6 minutes, with a median of 2.4 minutes, lower than the time found in a study conducted in Changhua (Taiwan)^4^ and in Daegu (South Korea)^5^, the average time of which was 33.1 minutes^4^ with a median of 68.5 minutes^5^. Additionally, studies conducted in Lübeck (Germany) and Scotland (United Kingdom)^3^ ^,^ ^19^ also collected smoke from electrocautery in the cut and coagulation modes; cut and coagulation modes may vary from surgery to surgery.

Note that there are more than 100 types of PAH in nature^8^, however, the liquid chromatograph used in this study detects only 16 types. Two PAH chemical compounds were identified in this study, naphthalene and phenanthrene. These compounds were found in the air of operating rooms in other studies conducted in Lübeck (Germany)^3^, Changhua (Taiwan)^4^ and in Uppsala (Sweden)^10^.

The PAH concentrations found in this study were similar to those found by the study conducted in Uppsala, in which naphthalene was more frequently (97.5%) identified in the surgical smoke collected from 40 peritonectomies, followed by phenanthrene (93%)^(10)^. In this study, naphthalene was not identified in two of the videolaparoscopic procedures (one cholecystectomy and one appendectomy), while phenanthrene was not detected in only one surgery (exploratory laparotomy). At least one of the compounds was present in all three surgeries.

The negative impact of PAH on human health, regardless of concentration, has already been acknowledged as having high carcinogenic potential, in addition to deleterious effects on the skin, liver and immune system^8^. Therefore, the naphthalene and phenanthrene airborne compounds identified in the operating rooms are harmful and pose a risk to the health of the intraoperative team.

Naphthalene was classified as potentially carcinogenic and, even though this effect was not reported in humans, but only in experimental rats, exposure through the inhalation of this compound arising from surgical smoke may be associated with various types of cancer in humans, such as in the lung, olfactory and nasal tissues^21^. Inhalation of this compound is also possibly associated with cataracts, fatigue, headaches, liver and kidney damage, in addition to hemolytic anemia in humans^12^.

Among the various effects concerning phenanthrene, we highlight: irritation of skin and respiratory tract; cough; sore throat; eye redness; and abdominal pain^11^. The carcinogenic potential of phenanthrene has not been established either in experimental animals or humans^9^.

Understanding of any cause and effect relationship in the development of pathologies in humans is still incipient, as studies^4^ ^,^ ^10^ report the presence of various PAHs in the air of surgical environments, which impedes relating any given compound with the specific development of a given pathology.

This study's results show a significant and strong correlation (0.761) between the amount of naphthalene and phenanthrene produced. Hence, we assume the production of these two compounds increases concomitantly.

The average concentrations of naphthalene (0.0053 mg/m^3)^ and phenanthrene (0.0007 mg/m^3)^ were higher than the averages reported by other studies. One study conducted in Changhua (Taiwan) with mastectomies found average concentrations of 0.001055 mg/m^3^ of naphthalene and 0.0000843 mg/m^3^ of phenanthrene^4^. The previously noted study in Uppsala (Sweden) addressing peritonectomies also identified lower average concentrations: 0.0001 mg/m^3^ of naphthalene and 0.00000627 mg/m^3^ of phenanthrene^10^.

PAHs occupational exposure limits are regulated by an American agency: 50 mg/m^3^ is the limit concentration for naphthalene and 0.2 mg/m^3^ for phenanthrene as an average for an eight-hour workday^11^ ^-^ ^12^. Exposure in this study was assessed per surgery rather than based on the duration of the daily exposure of each member of the intraoperative staff. For this reason, the PAH concentrations were not compared to the recommended occupational exposure limits, while none of the surgeries lasted more than eight hours.

Even small concentrations of these compounds are a concern, if we consider the frequent use of electrocautery and consequent cumulative effects. The intraoperative staff is exposed to small amounts of smoke compared to the other professionals, but the exposure is more prolonged and more constant^1^. Therefore, regardless of the PAH concentrations arising from surgical smoke, the effects are cumulative, similar to the cumulative effect of tobacco smoke, and are not necessarily identified^7^.

The adoption of preventive measures should be a priority in SC to minimize the chemical hazards to which these professionals are exposed in the inhalation of surgical smoke, including the use of PPE and the installation of local exhaust and efficacious ventilation systems in operating rooms^15^ ^-^ ^16^. Recommended measures to minimize the effects of electrocautery use include PPE. Respirator masks such as the N95 type filter at least 95% of aerosols, gases and fumes^17^. The use of respirator masks is regulated by an American occupational health agency as secondary protection against surgery smoke inhalation^15^. In this study, however, none of the members of the intraoperative team used any type of respirator. Note none of the patients undergoing surgeries were under airborne precautions.

The use of surgical or procedure masks only is common in many countries^22^ ^-^ ^23^. Surgical masks do not properly protect the intraoperative team against microorganisms or pathologies transmitted by the aerosol product, gases or fumes^17^ produced by the electrocautery. Even though this type of PPE is still recommended in Brazil, a small percentage (10%) of workers and graduate students neglect its use.

In addition to respirator masks, international regulations recommend safety goggles for the entire team exposed to surgical smoke^16^, but only three graduate students from the field of general surgery (5%) wore this PPE.

The current Brazilian regulatory standard - 32 (NR-32) - recommends the use of goggles to protect from exposure to biological fluids without mentioning the need for this PPE to protect against surgical smoke^24^.

The adoption of preventive measures - such as the use of local exhaust and ventilation systems together with PPE - is essential for the safety of the intraoperative team. These measures, allied with continuous education to sensitize the team, can minimize chemical hazards and consequently enable a safe and healthy work environment for all workers and graduate students.

This study's limitations include its cross-sectional design, which does not allow the results to be generalized, and the fact we did not assess an eight-hour workday exposure to surgical smoke was also a limiting factor and hindered comparisons with occupational levels recommended internationally. Thus, future studies are needed to obtain further scientific evidence.

## Conclusion

The operating rooms of the SC addressed in this study are not in agreement with either national or international standards, as they do not have local exhaust and ventilation systems to purify the air. 

PAH concentrations, naphthalene and phenanthrene, arising from surgical smoke produced by electrocautery, were identified in the air of the operating rooms in all the surgical events assessed, both procedures by incision that might be open and large and small, like videolaparoscopic procedures, while a strong correlation was found between the naphthalene and phenanthrene variables.

Total PAH concentrations, which ranged from 0.0061 mg/m³ to 0.208 mg/m³, indicate that workers and graduate students in the intraoperative team are constantly exposed to chemical compounds that are harmful to human health due to their cumulative effects.

Adherence to utilizing PPE, such as safety goggles, on the part of the intraoperative team is low and most use masks without filters, therefore, masks that are considered inappropriate for PAH exposure. In addition to personal protective measures, collective measures should be implemented to improve air quality in operating rooms.

This study supports future studies intended to identify harm caused to the health of workers and graduate students exposed to surgical smoke and to encourage a healthy and safe work environment for the intraoperative team.
